# UDCAD-DFL-DL: A unique dataset for classifying and detecting agricultural diseases in dragon fruits and leaves

**DOI:** 10.1016/j.dib.2025.111411

**Published:** 2025-02-19

**Authors:** Pronob Chandra Sarkar, Gourab Kumar Pranta, Mayen Uddin Mojumdar, Arif Mahmud, Sheak Rashed Haider Noori, Narayan Ranjan Chakraborty

**Affiliations:** Multidisciplinary Action Research Laboratory, Department of Computer Science and Engineering, Daffodil International University, Birulia, Dhaka 1216, Bangladesh

**Keywords:** Agriculture, Bacterial diseases, Computer vision, Dragon fruit, Leaf diseases, Sunburn damage, Stem canker

## Abstract

Leaf and fruit diseases are unavoidable due to the unpredictability of climate variability and environmental changes. This study presents dragon fruit and leaf diseases dataset. The dataset includes a total of 4,518 clear and detailed images. The dataset lists the following diseases of leaf and fruit along with the number of images for each: Bacterial Diseases (498), Fungal Infections (Anthracnose or Stem Canker) (84), Healthy Leaves (2,242), Sunburn Damage (271), Healthy Fruits (333), Insect Infected Fruits (53), Mealybugs and Scale Insects (Fruits) (173), Bacterial Wilt (Fruits) (84). All images were captured with two smartphones one is Google Pixel 7 Pro and another one is iPhone 14 Pro in the Dhaka Division. This dataset focuses on analysing the diseases of Dragon leaf and fruit to improving the agriculture sector.

Specifications Table

**Table:** 

Subject	Agriculture Sciences, Computer Sciences.
Specific subject area	Computer Vision, Image Processing, Image Classification, Machine Learning.
Type of data	The images are in JPG format (total 4518 jpg files) having 3024 × 4032-pixel dimensions and 96 × 96 dpi image resolution.
Data collection	Given dataset represents a total number of 4518 raw images which contains dragon leaf and fruit diseases. The images were taken with two smartphones one is Google Pixel 7 Pro and another one is iPhone 14 Pro.
Data source location	Amin Model Town, Ashulia, Savar, Dhaka Division, Bangladesh (Latitude: 23° 54′ 26.0″ N, Longitude: 90° 16′ 00.7″ E)
Data accessibility	Repository name: Mendeley Data Data identification number: DOI:10.17632/cfchfdpfw5.1 Direct URL to data: https://data.mendeley.com/datasets/cfchfdpfw5/1

## 1 Value of the Data

• The dataset serves as an important educational resource for farmers and agricultural related workers. It provides clear information about various diseases. For providing our dataset it will helps or allows farmers to make better informed decisions and farmers can implement more efficient farming techniques.
• Dragon fruit's demand is increasing day by day because of its health benefits. So, it is important to keep crops in good condition so that communities can enjoy this essential food. Our dataset can play a direct rule in securing fruits and public health.
• This dataset is essential for developing early warning systems focused on dragon fruit and leaf diseases. For this system researchers can predict what might happen in the future by reviewing last data. And it will help reduces crop losses and more stable production.
• Researchers can effectively work on breeding dragon fruit varieties because of our dataset availability. So, it is making a significant contribution to generic research support.
• Through our dataset the information will be distributed globally and supporting farmers across different regions in upgrading their farming techniques. This united effort encourages the exchange of ideas. In the end, it contributes to the long-term sustainability of agriculture.

## 2 Background

The motivation for working with this dataset on dragon fruit plants is the increasing need for precise monitoring of plant health and managing crop diseases effectively [1]. There is huge demand for dragon fruit nowadays; for that reason, it is a significant crop with both economic and nutritional value. This fruit needs proper care for its growth because it is a desert plant that can survive in rough conditions. But as a country of Asia, Bangladesh is full of natural resources with fertile land. For that we try to monitor the condition of this plant to help in the agricultural side and use the machine learning and computer vision technologies to monitor this plant's health [4]. In this dataset we try to collect all kinds of necessary images of dragon fruit plants, such as those affected by bacteria, fungi, and insects, or by birds, but most of the images are of healthy leaves and fruits. We collect the data at different time periods, but most of the data are collected at noon and in the afternoon because at that time the proper light conditions are available, and we capture the perfect as required. And for that, we try to collect as much as we can [12]. This dataset was created to fulfil the need for supporting research initiatives in agriculture and environmental science. The main goal is to support the creation of an AI system for identifying, detecting, and recognizing dragon fruit and leaf diseases. This dataset serves catalog of bacterial wilt, fungal diseases like anthracnose, insect infections in dragon fruit farming [2,3]. This research also aligns with the recent trends in fruit and leaf disease detection using automation, which integrates image processing, machine learning and environmental studies. These technologies will contribute to solving some of the challenges created by conventional methods of classification, which cannot differentiate between visually similar yet subtly different symptoms of the disease. In addition, the dataset supports the overall efforts in biodiversity evaluation and protection by uncovering essential insights into the health of dragon fruit and leaf.

This dataset helps in different fields like researchers, agriculturists, and AI practitioners, improving plant disease management, crops, and agricultural allowances. This dataset helps to classify healthy and diseased leaves and fruits. This dataset also helps to observe the local service status with modern machine learning tools, which create an impactful harmony between machines and old age studies, which are much more time-consuming and contain huge amounts of information.

## 3 Data Description

**Fig. 1:**
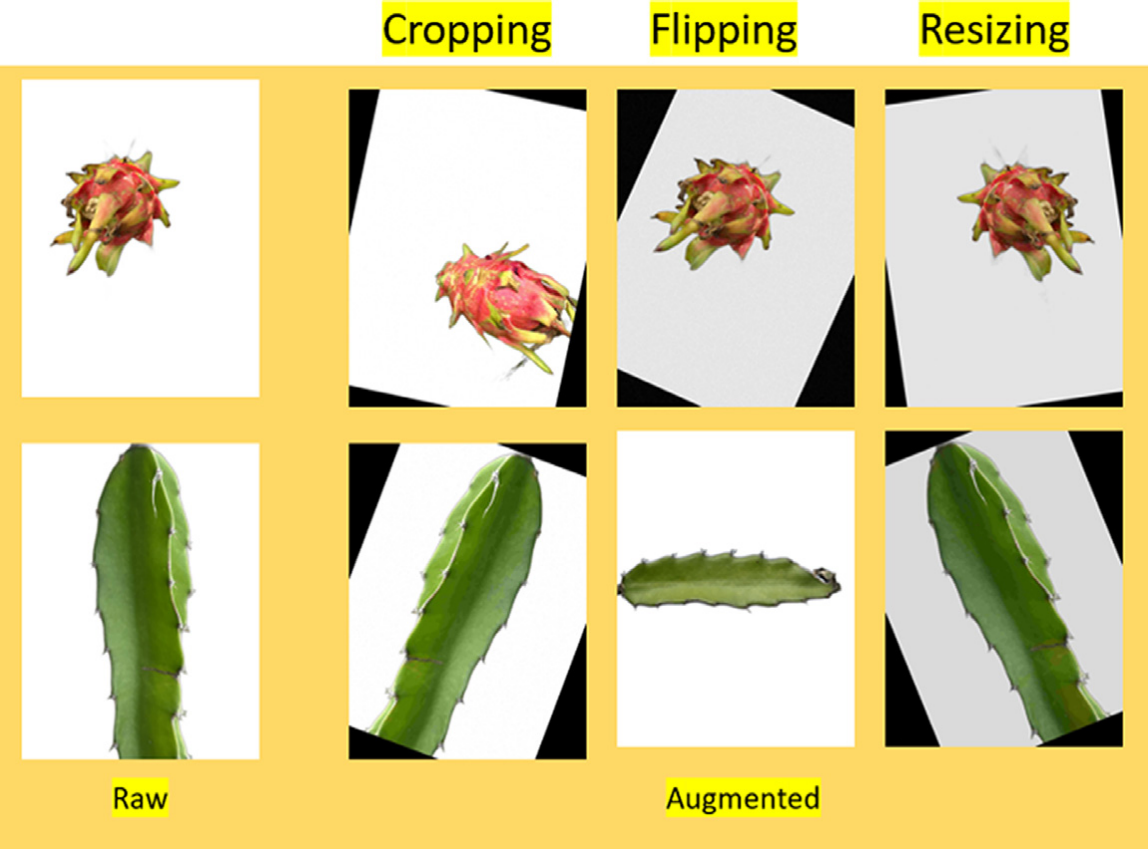
Picture of Raw and Augmented image.

This dataset is a collection of pictures aimed at testing ML and CV techniques for recognizing different variants of dragon fruit as well as dragon leaf with different kinds of diseases. The dataset shows different types of data that we collect for my work. We collect different types of data, like healthy and unhealthy, and then classify them in different classes as they should be. The primary challenge is to collect that data. As we take the picture one by one, it takes a good amount of time to collect the total number of images. We used two different devices to collect those images; one is an iPhone, and the other one is a Google Pixel 7. Each device was good for collecting the data. We used a white paper to remove the outside noise of nature and take the perfect image. Also, the dragon fruit's growth is faster than other fruits; for that reason, we try to collect the image and work with these fruits because now these fruits are popular in our country. The demand for these fruits is increasing day by day. And people are also showing interest by gardening and so on; the market value is quite good among other fruits. In this modern era, it is now common to have automation in every sector, and in Asian countries, agriculture is quite popular, and so this automation in agriculture is a game changer that benefits a lot to the farmers and also to the economy. In his two houses, it is quite costly to maintain human work as a worker. Instead of this human, if automation takes place, then it is quite easier and also faster because doing the same kind of job automation was built for. For this reason, we thought to take this as a plus point because a huge number of fruits is available in the season, and for this, use machines for the fruits and the leaf disease detection that will be helpful to the farmers and the workers to reduce costs. Also, machines are more consistent in work than humans, so for productivity, this is very helpful use of the automation system. In my dataset, all the fruits are the same: red dragon fruit. The reason for selecting this particular bread is that it is the most popular among others. Also, it is available in local fruit shops and gardens compared to other bread. The dataset includes a picture of a dragon fruit and its leaf suffering from specific diseases in different conditions to ensure measurement in a broad scope of contexts. Prepared to explain the contexts, this dataset is an essential tool for researchers and professionals in the fields of botany, agriculture, and AI. This investigation is based on a large dataset containing images that are in JPG format, totalling 4518 images, all of which are in JPG files, having 3024 × 4032-pixel dimensions of original images and 13,554 augmented images of dragon fruit and leaf, highlighting variations in backgrounds, lighting conditions, and stages of disease progression. Data augmentation is the method used in machine learning for increasing the amount of data in a dataset through alterations of the currently available data points. This approach works well for a model's generalization abilities in scenarios where there is limited available data so as to mitigate the chance of overfitting shown in Fig. 1. As a first step in the augmentation process, specific objectives are first defined, for instance, to guard against rotation invariance, guarantee invariance to small changes in lighting, or achieve invariance to change in scale of the objects.

The systematic application of transformations through data augmentation helps the models to learn better from the available data. The methods that are to be used for data augmentation include rotation of images, flipping the images horizontally or vertically, cropping, resizing, translating, and changing the features of the images, such as brightness, contrast, and colour saturation [11]. The sophisticated augmentation could include adding noise, elastic transformation, or cutout for simulation of occlusion. Most of the frameworks, such as TensorFlow and PyTorch, do a great job of providing excellent ways to use techniques of data augmentation with their libraries. Augmented data used during training fortifies the models to deal with changes and work well with different kinds of inputs. That would eliminate the need for more data to be collected to enlarge the dataset. That is important since overfitting risks may be raised when dependence on small datasets increases. Data augmentation enables models to learn the patterns in a better way and avoid problems related to little data. The model will be more accurate but also perform well on new data in real-life situations. Finally, data augmentation is an inexpensive method to make the most out of the data at hand; this leads to more reliable and flexible machine learning applications. This method allows machine learning to automatically identify dragon fruit and leaf diseases. As a result, this dataset offers a strong foundation for image-based classification and detection methods [[7], [8]]. We classified our dragon leaf images into a total of four classes after collecting the images as their condition of the leaves as shown in Table 1.

**Table 1:** Statistics of the dragon leaves dataset.

SL	Diseases Name of Leaf	Number of Images	Augmented Images
1	Healthy Leaves	2242	6726
2	Bacterial Diseases	498	1494
3	Sunburn Damage	271	813
4	Fungal Infections (Stem Canker or Anthracnose)	84	252

In Table 2, we provide a detailed breakdown of the Dragon fruits of the dataset, including the number of original and augmented images of the image that collect as raw images and then categorize in different classes based on the condition of the fruits.

**Table 2:** Statistics of the dragon fruit dataset.

SL	Diseases Name of Fruit	Number of Images	Augmented Images
1	Healthy Fruits	333	999
2	Insect Infected Fruits	53	159
3	Bacterial Wilt	84	252
4	Mealybugs and Scale Insects	173	519

For all these different classes of data, we merged them into four different classes. Healthy leaves and all the other leaves that are diseased categories are in the class unhealthy leaves. We do the same for the fruit data. Healthy fruits and all the diseased fruits are in the unhealthy fruits class, as shown in Table 3, where we also compare my dataset with other datasets available in Mendeley.

**Table 3:** Comparison table of 4 classes.

Class	Description	My dataset	Morales dataset [17]	Vedant dataset [16]	Nirob's dataset [15]
Healthy Leaves	Includes all healthy leaf images	2242	300	X	X
Healthy Fruits	Includes all healthy fruit images	333	X	1315	3779
Unhealthy Leaves	All diseased leaf categories (e.g., bacterial, fungal, sunburn damage)	1633	300	X	X
Unhealthy Fruits	All diseased fruit categories (e.g., bacterial wilt, insect-infected)	310	X	1248	X
Total	–	**4518**	**600**	**2563**	**3779**

In our dataset we try to include all the necessary information we can; by doing that, the uniqueness of our dataset increases compared to other datasets that are available, we remove the background of our data so that the data we are working with can clearly allocate in Table 4 we try to show the difference of our dataset with others datasets where the difference of the number of collected images and others are shown.

**Table 4:** Comparison table of different dataset.

Dataset Name	Total Images	Classes	Uniqueness	Sample of Data
Our dataset	**4518**	Healthy Leaves, Healthy Fruits, Unhealthy Leaves, Unhealthy Fruits	We try to include the default condition of leaves and fruits like sunburn and bacterial wilt and others type	
Morale's dataset [17]	**600**	Sick plants, Healthy plants	Focused on plants only with background noise	
Vedant dataset [16]	**2563**	Multiple class of Fruits	The growth of raw dragon fruits and ripe dragon fruits background natural interferences	
Nirob's dataset [15]	**3779**	Multiple class of Fruits	Collect the mature, immature, fresh and defect fruits	

We collect various types of data on dragon fruit leaves and fruits. Table 5 presents an overview and details of the dataset. We take help from an agronomist, Mr. Abdul Mannan Mojumdar, who gives us proper guidelines, and we follow them to detect the proper class according to the diseases.

**Table 5:** Dragon leaf and fruit diseases picture with their name.

Class	Description	Sample Images
Healthy Leaf	There are two species of health dragon fruit plants: Hylocereus undatus and Hylocereus costaricensis. Healthy dragon fruit leaves are thick, fleshy and deep green in color. They usually have serrated or wavy edges, it helps capturing sunlight for photosynthesis. If the surface is smooth then it indicates good hydration and good nutrient states [6].	
Bacterial Diseases	Dragon fruit plants (Hylocereus spp.) are significantly at risk to bacterial infections which mainly affect their leaf structures. Bacterial Canker (Pseudomonas syringate), Bacterial Blight (Xanthomonas), Bacterial Soft Rot (Erwinia carotovora) and Leaf Spot disease are the different stages of Bacterial diseases of Dragon Fruit plant leaves [6].	
Sunburn Damage	In Bangladesh, dragon fruit trees frequently suffer from sunburn damages, particularly during summer months when temperature rise beyond 38 °C. from This problem usually occurs in March and April, when there are notable variations in the daytime and nighttime temperatures. Because the stems lose clorophyll when exposed to high amounts of sunshine, the plants may seem bleached. The western side of the plant stems may be 10 % to 50 % sunburned [[10], [13]].	
Fungal (Anthracnose or Stem)	Fungal diseases are mazor problem in most plantation crops in Bangladesh. Some recorded fungal diseases observed on dragon fruit plants are stem end rot, brown spot, anthracnose. But Stem Canker is one of the most destructive diseases in dragon fruit plantaion. The symptoms can be observed as circular, brown sunken lesions with white mycelium formation on the lesion surface. Fusarium proliferatum, Xanthomonas campestris, F. oxysporum or Erwina caratovora are all plant pathogens that can affect dragon fruit plantations (pitaya). Environmental changes also reason for fungal infection increase [6].	
Insect Infected Fruit	Dragon fruit plants are susceptible to damage caused by both insects and birds. This damage can significantly affect fruit quality and yield [5].	
Mealybugs and Scale Insects Fruit	Mealybug (Pseudococcidae) insects consume the plant's sap. Consuming sap will reduce the plant's growth. There are two kinds of scale insects: armored Scale (Diaspididae) and soft Scale (Coccoidea). Soft Scales produce honeydew that can cause sooty mold to grow on plant surfaces. Photosynthesis may be hampered by this. Armored Scale is a kind of insects that has hard outer shell. This shield protects these insects from pesticides and natural predators. So, it has become very difficult to manage these insects [[5], [9]].	
Bacterial Wilt Fruit	Bacterial Wilt in Dragon Fruit is a serious disease caused by the bacterial pathogen Ralstonia solanacearum. It affects the vascular system of the dragon fruit plant, leading to significant damage and, in severe cases, plant death. Below are the details regarding the disease [6].	
Good Fruit	Depending on the variety, fresh dragon fruit has a bright skin that ranges in color from pink (Hylocereus costaricencis), red (Hylocereus polyrhizus), and sometime yellow (Hylocereus megalanthus). The skin is usually covered with scales or spikes for that it creates unique look. On the inside, the flesh is white or red as well as soft and juicy. When we pick a dragon fruit [5].	

**Fig. 2:**
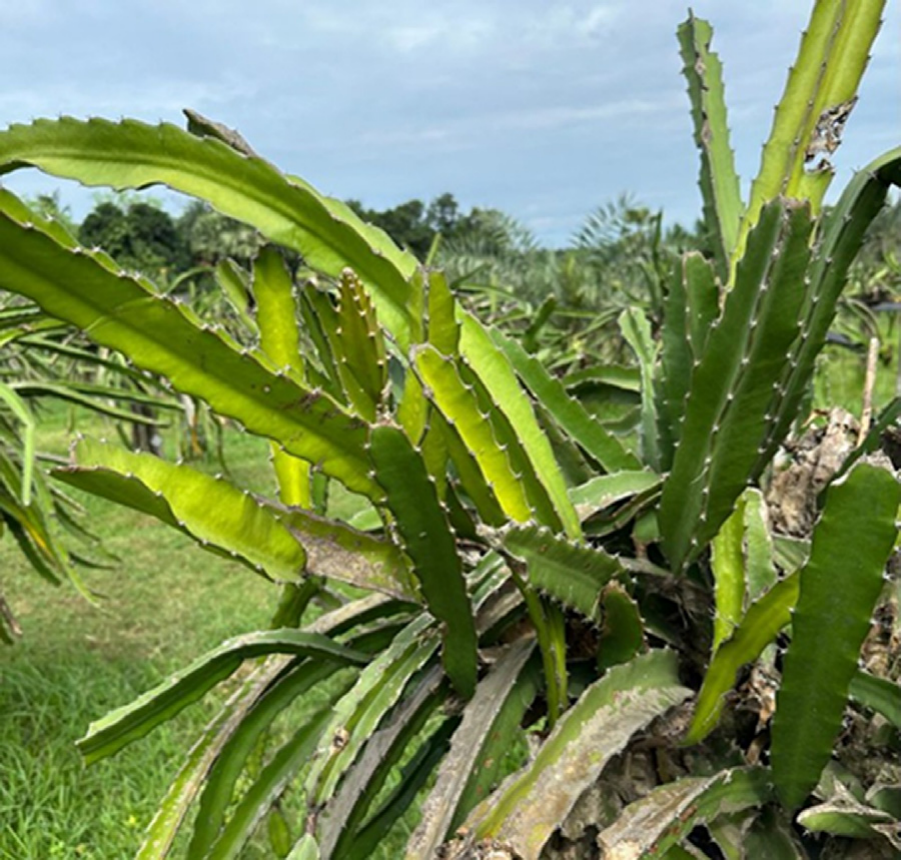
Picture of field.

We collect our data throughout October and November 2024. We captured the leaf and fruit images on different days and at different times of the day. We collect the image data from Fiber Plus Agro, which is located near Daffodil International University, Ashuliya, Savar, Dhaka, Bangladesh. In Fig. 2, showing the real-time image of the location. We collect all the images on different days and different time periods, which help me collect the data and observe the fruit and leaf for a couple of days. At that time, we observed that the growth of the fruit was quite faster than other fruits and also attracted the insects and birds with its red color. For that reason, farmers use cover on the ripped and semi-ripped fruits for maintaining their growth and their own protection from the insects [14]. Location was the same, so that helped us to collect all the data easily. Details are given in Table 6.

**Table 6:** Collection details of dragon leaf and fruit dataset.

Class Name	Weather	Date	Time	Temperature (°C)	Camera Device	Location
Insect Infected Fruit	Sunny	27 October 2024	Morning	27 °C	iPhone 14 Pro	Amin Model Town, Ashulia, Savar, Dhaka Division, Bangladesh (Latitude: 23° 54′ 26.0″ N, Longitude: 90° 16′ 00.7″ E)
Mealybugs and Scale Insects Fruit	Windy	27 October 2024	Afternoon	26 °C	Google Pixel 7 Pro and iPhone 14 Pro	
Healthy Leaf	Sunny	29 October 2024	Morning	31 °C	iPhone 14 Pro and Google Pixel 7 Pro	
Bacterial Wilt Fruit	Sunny	28 October 2024	Noon	30 °C	Google Pixel 7 Pro and iPhone 14 Pro	
Good Fruit	Cloudy	21 October 2024	Morning	29 °C	Google Pixel 7 Pro and iPhone 14 Pro	
Sunburn Damage	Cloudy	03 November 2024	Afternoon	27 °C	iPhone 14 Pro	
Fungal (Anthracnose or Stem)	Cloudy	03 November 2024	Morning	29 °C	iPhone 14 Pro	
Bacteril Diseass	Cloudy	05 November 2024	Afternoon	28 °C	iPhone 14 Pro and Google Pixel 7 Pro	

## 4 Experimental Design, Materials and Methods

### 4.1 Preprocessing

**Fig. 3:**
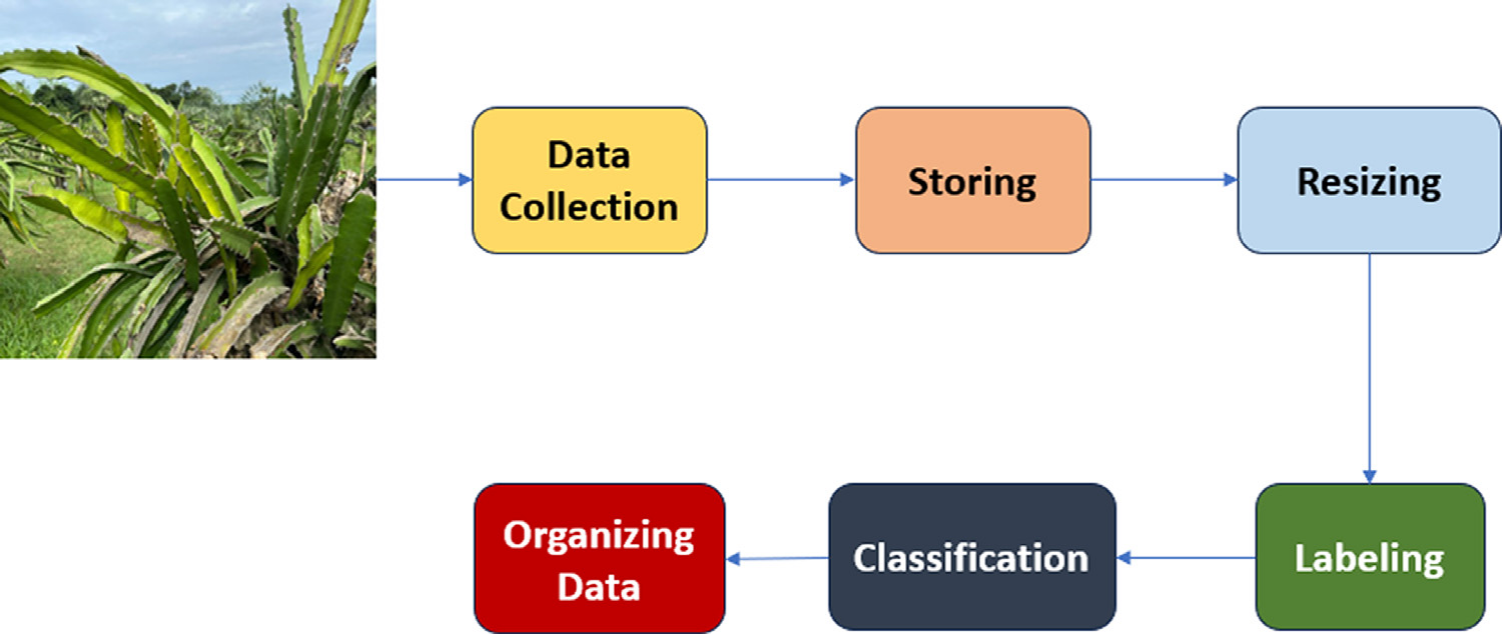
Process of steps of the whole workflow.

A detailed preprocessing workflow was developed to prepare the dataset to achieve stability and upgrade its suitability for processes such as resizing, data augmentation. Initially, images are sourced from a specific directory and loaded into the system's memory. The (rembg) is used to remove backgrounds, which highlights the main subjects (dragon fruit leaves or fruits) on a transparent screen. The photos that are taken by iPhone are in HEIC format. To convert those to JPG, we use a function that takes two parameters: one is the image path, and the other one is the temporary path, which is for saving the converted image. Then the (pillow_heif) reads the iPhone image path and converts the image into JPG by using the (pillow_heif) function. Also, there are different free sites that are also available that can convert the HEIC file to JPG form them we used iloveimg.com sites to convert the pictures that were taken by iPhone. At the background removal point, every image is converted to the RGBA format. To achieve clarity in how the dataset is visually presented, the transparent sections are filled with a solid white background, which helps to highlight the subject in each image. After this step, the images are converted to RGBA format, which removes the alpha layer and confirms that all images are prepared in a consistent style for the machine learning task. All images in this dataset were captured using smartphone cameras from the same distance from the subject, with proper focus and zoom. After this, a total number of 4518 images of dragon fruits and leaves were captured. The overall working process is given in Fig. 3. Every image was taken in natural light; also, apply a white paper background to remove environmental noise and limit distractions from the background. These images were captured in different environmental conditions. Furthermore, pictures were taken at different times of the day to enhance image quality in daylight. The dataset was compiled from approximately 900 dragon trees located in a garden near the permanent campus of Daffodil International University in Ashulia, Savar, Dhaka. After processing the images, they are stored in a specific output directory, ready for resizing and data augmentation. The dataset stability and all variations that may arise because of lighting, environmental conditions, or irregularities are minimized.

This preprocessing flow ensures that the dataset is significantly prepared for impactful learning tasks, especially in the identification analysis of disease on dragon fruits and leaves.

## Limitations

The significant shortcoming of this dataset is geographic, the base images of the dataset were collected from certain regions with specific environmental conditions. So, there is regional bias, which might limit the ability of the dataset to represent the variability of the dragon fruit types and their health status in other geographic or ecosystem conditions. Besides that, the conditions of light, weather, and background differ every time a capture of an image is done. Such inconsistencies can reduce the general quality and uniformity of the images, thus probably decreasing the accuracy with which plant health is classified. Critical features of diseased or healthy plant parts may be obscured in images that are overly bright or shadowed. This variability underlines the difficulties in achieving consistent image quality in the outdoors. Another major concern is the seasonal limitation of the methodology for collecting data. Since images were taken only during specific periods of the year, the dataset may not represent health conditions or other botanical activities occurring outside of those periods. The presence of this seasonal bias could limit its use for the active monitoring of dragon fruit health throughout the whole year [12].

## Ethics statement

The authors declare that this research does not involve human subjects or animal experiments, nor the use of social media data. Data for this study are field-based and consisted of observations of flowering species in the wild. The authors also assure that the current work conforms to different ethical aspects for publication in Data in Brief. This research does not involve human participants as data were obtained from Twitter and all the animals involved in the study were acquired from one of the linked studies that have been previously published with proper ethical approval; therefore, applicable informed consent, animal handling or social media data redistribution policies are irrelevant here.

## Credit Author Statement

**Pronob Chandra Sarkar**: Writing - Original Draft, Data Curation; **Gourab Kumar Pranta**: Visualization, Writing - Review & Editing; **Mayen Uddin Mojumdar:** Methodology, Supervision**; Arif Mahmud**: Writing - Review & Editing; **Sheak Rashed Haider Noori**: Software; **Narayan Ranjan Chakraborty:** Conceptualization.

## Data Availability

Mendeley DataDragon fruit & leaf Dataset from Bangladesh for Classification and Ecological Research (Original data).

## References

[1] Zhang K., Wu Y., Li X. (2023). Automated plant disease detection and classification using deep learning approaches: a case study on agricultural crops. Agric. Informatics Res. J..

[2] Roy T., Islam M., Das S. (2022). Transfer learning-based approach for leaf disease detection in tropical plants. IEEE Trans. Image Process..

[3] Shinde M., Chaudhari H.B., Patil S.T. (2023). A comprehensive survey on the role of AI in crop disease prediction and monitoring. Comput. Electron. Agric..

[4] Chen J.F., Wang X., Yu T.Q. (2024). Deep neural networks for fruit disease detection in smart agriculture applications. Sensors Actuators B: Chemical.

[5] Bhattacharya S.K., Nath P., Banerjee R. (2023). IEEE International Conference on Advances in Computing and Communication (ICACC).

[6] Pham H., Nguyen L. (2023). Detection of bacterial and fungal diseases in tropical fruits using computer vision techniques. J. Trop. Agric. Res..

[7] P. Das, A. Mishra, and D. Sahoo, “Combining CNN and transfer learning for classifying dragon fruit plant health conditions,” Springer Advances in Agricultural Informatics and Systems Biology, pp. 112–128, 2023. doi: 10.1007/s11042-023-13246-1.

[8] Singh A., Choudhary B. (2024). Exploring lightweight CNN models for real-time disease detection in fruits. Comput. Agric. J..

[9] Silva D.E., Kumar A.G. (2023). Proceedings of the 2023 IEEE International Conference on Smart Agriculture (ICSA).

[10] Alvarez L., Ortega T., Perez M. (2023). Classification of sunburn damage in dragon fruit using multispectral imaging and Deep learning. J. Precision Agric..

[11] BOZKURT F. (2022). A study on CNN based transfer learning for recognition of flower species. Eur. J. Sci. Technol..

[12] Chen J.F., Wang X., Yu T.Q. (2024). Deep neural networks for fruit disease detection in smart agriculture applications. Sensors Actuators B: Chemical.

[13] Mojumdar M.U., Chakraborty N.R. (2021). 2021 12th International Conference on Computing Communication and Networking Technologies (ICCCNT).

[14] Dutta M. (2024). Rice leaf disease classification—A comparative approach using convolutional neural network (CNN), cascading autoencoder with attention residual U-net (CAAR-U-Net), and MobileNet-V2 architectures. Technologies (Basel).

[15] Khatun, Tania; Nirob, Md.Asraful Sharker; Bishshash, Prayma; Uddin, Mohammad Shorif (2023), “Dragon fruit maturity detection and quality grading dataset”, Mendeley Data, V1, doi: 10.17632/2jpzbx8tm6.1. 10.1016/j.dib.2023.109936 PMC10733099 38125368

[16] C. Singh, P. Ingle, V. Modi, P. Kosamkar, V. Kulkarni, June 18, 2022, “Dragon Fruit image dataset”, IEEE Dataport, 10.21227/7c17-7112.

[17] Gabriela Morales Soto, May 2, 2024, “Dragon Fruit plant image dataset”, IEEE Dataport, 10.21227/370q-5925.

